# Good Diagnosis

**Published:** 1883-08

**Authors:** 


### GOOD DIAGNOSIS.

It seems that New Orleans has been enlivened by the dean of a Medical College there, who diagnosed and treated an ulcer of the gums about a young lady's tooth as cancer, and having failed the case passed to a dentist, who extracted the tooth and let the "cancer" get well. A true victory for nature.—*St. Louis Med. Jour.*

